# Dynamic Thymic Stromal Lymphopoietin and Its Receptor Complex Expression on Skin Langerhans Cells in Response to MC903‐induced Skin Inflammation

**DOI:** 10.1002/eji.70178

**Published:** 2026-03-30

**Authors:** Martín I. González‐Rodríguez, Tanja Salomaa, Carolina Cabalin, Tuomas Komulainen, Lotta Hiihtola, Laura Kummola, Tero A. H. Järvinen, Ilkka S. Junttila

**Affiliations:** ^1^ Faculty of Medicine and Health Technology Tampere University (TUNI) Tampere Finland; ^2^ InverSkin SpA Santiago Chile; ^3^ Department of Infectious Diseases and Pediatric Immunology School of Medicine, Santiago Pontifical Catholic University of Chile Santiago Chile; ^4^ Department of Dermatology Weill Cornell Medicine New York New York USA; ^5^ Department of Orthopedics and Traumatology TAUH Tampere Finland; ^6^ Fimlab Laboratories Tampere Finland; ^7^ NordLab Oulu Finland

**Keywords:** Langerhans cells, TSLP

## Abstract

Thymic Stromal Lymphopoietin (TSLP) regulates the skin microenvironment during type 2 inflammation. Epidermal APCs, Langerhans cells (LCs), might be the target of TSLP, but whether LCs express CD127 needed for TSLP signaling has been unclear. We found that LCs express both receptor chains needed by TSLP (CD127 and TSLPR) and upregulate the TSLPR upon Calcipotriol (MC903)‐induced inflammation.

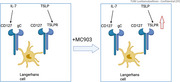

**Abbreviations**
AD: atopic dermatitis
DC: dendritic cell
IHC: immunohistochemistry
IL: interleukin
LC: Langerhans cell
MC903: Calcipotriol
RT‐qPCR: real‐time quantitative polymerase chain reaction
sDC: splenic DC
t‐SNE: t‐distributed stochastic neighbor embedding analysis
TSLP: Thymic Stromal Lymphopoietin

Langerhans cells (LCs) are specialized epidermal immune cells. They are critical initiators of immune response, which is further modulated by the cytokine microenvironment. LC diversity also promotes differential immune responses [1, 2]. In allergic inflammation, epithelium‐derived interleukin (IL)‐25, IL‐33, and Thymic Stromal Lymphopoietin (TSLP) modulate inflammation [1, 3, 4, 5]. TSLP may also have a regulatory function by inducing Treg differentiation through dermal dendritic cells (DCs) [6]. TSLP signaling requires expression of two cell surface receptor chains: IL‐7Rα and TSLPR (Figure S1A). Murine splenic or lymph node steady state DCs are not considered to express IL‐7Rα [7], but DCs activation upregulates IL‐7Rα expression in vitro [8]. In LCs, the expression of TSLPR has been studied [9], but IL‐7Rα expression remains enigmatic.

To learn if LCs are TSLP targets, we enriched LCs (CD207^+^/CD11c^+^/MHC‐II^+^) from unmanipulated mice (Figure S1B) and assessed IL‐7Rα (CD127), TSLPR, and CD132 (γC) expression (Figure 1A). LC IL‐7Rα expression was compared to T cells (IL‐7Rα^high^), B cells (IL‐7Rα^neg^), and splenic DCs (sDCs) (IL‐7Rα^neg^) [7] (Figure 1A,B and Figure S1B). LCs expressed higher levels and percentage of IL‐7Rα than did the sDCs (Figure 1A,B, *p* < 0.0001). TSLPR and γC were similarly expressed by sDCs and LCs (Figure 1A). We next assessed if in vivo TSLP induction [1] affected receptor expression. Mice's skin was subjected to Calcipotriol (MC903) twice (0 and 24 h), followed by euthanization at 48 h (Figure S1C). TSLP induction was measured by both real‐time quantitative polymerase chain reaction (RT‐qPCR) and immunohistochemistry (IHC) (Figure S1D,E, *p* = 0.004). TSLP receptors were measured by flow cytometry (Figure 1C,D) on LCs 48 h post‐treatment. IL‐7Rα expression remained unchanged (Figure 1C), but the GMFI of TSLPR expression was upregulated by MC903 (Figure 1C, *p* < 0.001). Since TSLP signaling requires both TSLPR and IL‐7Rα, we measured double‐positive cells. TSLPR^+^IL‐7Rα^+^ cells increased in the LCs from the MC903‐treated mice compared to the control (mean 46.27% vs. 19.42%, Figure 1D, *p* < 0.001, Figure S1F). Although TSLP has been shown to induce maturation and migration of dermal DCs [5, 6], the functional effect on LCs is unclear. We show that steady state murine LCs can respond to TSLP and IL‐7, as they express IL‐7Rα, TSLPR, and γC.

**FIGURE 1:**
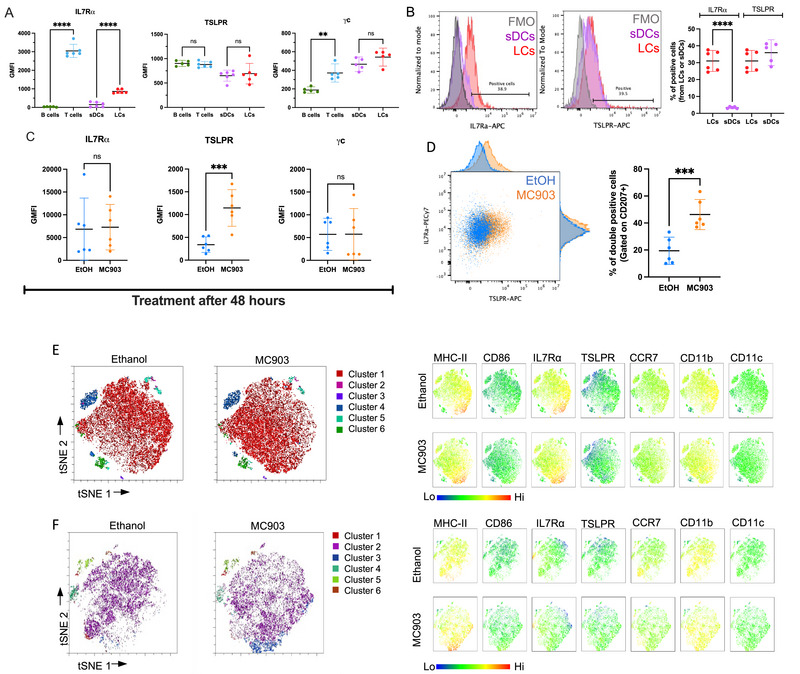
(A) Interleukin (IL)‐7Rα, TSLPR, and γC expression on Langerhans cells (LCs), splenic DCs (sDCs), B and T cells. GMFI indicated. (B) IL‐7Rα and TSLPR expression between LCs and sDCs with FMO controls and the quantification of positive cells (%). (C) IL‐7Rα, TSLPR, and γC expression on LCs after either MC903 or control 48 h post‐treatment. (D) IL‐7Rα and TSLPR expression on LCs in MC903 or control mice. Double‐positive cell quantification indicated. (E) t‐Distributed stochastic neighbor embedding (t‐SNE) analysis of LCs from control or MC903‐treated animals at 48 h (E) and 72 h (F). Markers in each cluster are indicated (next to t‐SNE). Data shown as scatter plots with mean as a bar and 95% confidence interval (CI) (*n* = 6 mice per group, two independent replicas). Analyzed with one‐way ANOVA with Tukey post‐test comparison, **p < 0.01, ***p < 0.001, and ****p < 0.0001.

LCs consist of different small subsets [2, 10]. To examine the LC subsets' response to MC903‐induced TSLP, we expanded the surface marker panel (Table S1) and follow‐up time. We analyzed LCs using t‐distributed stochastic neighbor embedding (t‐SNE) clustering 48 (Figure 1E) and 72 h post‐treatment (Figure 1F). Most cells belonged to one large cluster at both time points (Cluster 1, Figure 1E; Cluster 2, Figure 1F). 72 h post‐MC903 treatment, a cluster of cells (Cluster 3) was enriched (∼4% in control vs. ∼12% in MC903) with MHC‐II, CD86, and CD11b expression elevated (Figure 1F). While not identified as an independent cluster, neighboring cells with high expression of CCR7 were reduced, suggesting these LCs might be migrating away from skin (Figure 1E,F). The upregulation of MHC‐II and CD86 on LCs (Cluster 3, Figure 1F) matches the activated/migratory LC subset in humans [2].

We next measured IL‐7Rα and TSLPR in skin by double fluorescent IHC (Figure 2A and Table S2) or by IHC (Figure 2B). The receptor messenger RNA (mRNA) expression was also measured by RT‐qPCR in skin biopsies (Figure 2C). IL‐7Rα^+^ cells were rare in murine epidermis; IL‐7Rα expression was observed in CD207^+^ cells in the epidermis after MC903 treatment (Figure 2A and Figure S2A–E). IL‐7Rα upregulation is located mostly in CD3^+^ cells (Figure S3 and Table S2). TSLPR upregulation in the epidermis was observed by IHC in the MC903‐treated mouse skins (Figure 2B). No differences were found in mRNA expression from the whole skin samples (Figure 2C). Murine MC903‐induced TSLP expression induces similar clinical manifestations as seen in atopic dermatitis (AD) patients [1]. We evaluated the role of TSLP induction on its receptor complex expression in the context of pathogenesis and severity of AD in humans. We assessed the expression of TSLP RNA in patients’ tape strips, comparing non‐lesional, mild, moderate, or severe AD (Figure 2D and Demographic and clinical details in Table S3). We also evaluated the RNA and protein expression of TSLPR using RT‐qPCR and IHC (Figure 2E,F). We observed upregulation of the TSLPR gene in the epidermis of the skin biopsies from patients with moderate and severe AD (Figure 2E). The increased TSLP RNA expression is positively associated with higher TSLPR mRNA expression (Figure 2E). Similarly, mRNA for IL‐7Rα was upregulated in lesional AD with a tendency to be highly expressed in moderate and severe lesional skin compared to non‐lesional skin from tape strip samples containing the upper epidermal layer (Figure 2D). In contrast, TSLPR and IL‐7Rα protein seem to be highly expressed in the dermis in moderate‐severe skin biopsies compared to non‐lesional and moderate AD skin, as well as the expression of CD207^+^ cells (Figure 2F). Our results in AD patients indicate an upregulation of the TSLP and TSLPR in the epidermis of severe lesional skin consistent with our murine findings. To our knowledge, the high expression of TSLPR and IL‐7Rα proteins in the dermis of moderate to severe AD biopsies, compared to non‐lesional and mild AD skin, has not been described.

**FIGURE 2:**
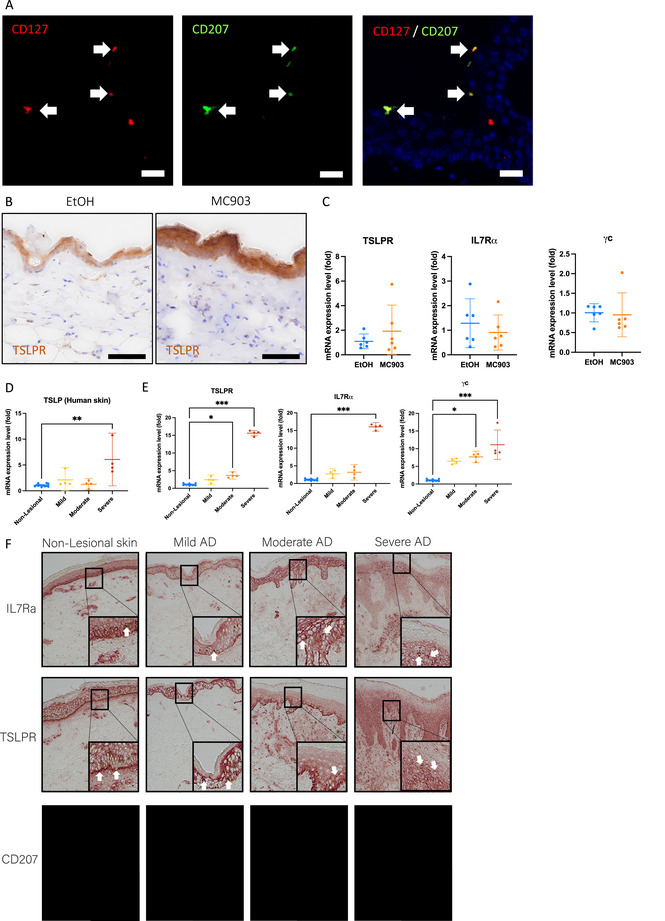
(A) Representation of epidermal CD127^+^/CD207^+^ cells 48 h after MC903 treatment by double immunofluorescent immunohistochemistry (IHC), bars represent 20 µm, arrows indicate double positivity. (B) TSLPR expression in mouse skin using IHC, representative images shown, bars represent 50 µm. (C) Expression of receptor chain messenger RNA (mRNA) by real‐time quantitative polymerase chain reaction (RT‐qPCR) in mouse skin. (D) Expression of TSLP and (E) TSLPR, IL‐7Rα, and γC from patient‐skin tape strips using RT‐qPCR (data analyzed using fold change [2^‐ddCt^]). Representative IL‐7Rα, TSLPR, and CD207 stainings in non‐lesional, mild‐, moderate‐, or severe atopic dermatitis (AD) by IHC (F). Data shown as scatter plots with mean as bar and 95% confidence interval (CI) (*n* = 6, two independent replicas for mice; *n* = 4 for different AD patient groups, 12 for non‐lesional skin). Analyzed with *t*‐test or one‐way ANOVA with Tukey post‐test comparison, ***p < 0.001.

Although LCs share many functions with DCs, they are closer to macrophages in cell development and ontology [3], and comparing sDCs with LCs might be artificial. Furthermore, TSLP induction may trigger both inflammatory and regulatory signals affecting skin homeostasis, including dermal DCs and ILC activation [10]. Evaluating the potential regulatory functions LCs may have, our findings would need to be evaluated in an environment where the TSLP function could be blocked.

TSLPR and IL‐7Rα on LCs may enable rapid responses to early immune signals, while their differential expression in LCs compared to sDCs suggests niche‐specific cytokine regulation. In steady state, IL‐7Rα on sDCs would sequester IL‐7 from T cells in the spleen, but on skin where IL‐7 expression is low, such interference would not occur. This differential expression might reflect tissue‐specific cytokine responsiveness.

## Author Contributions

**Martín I. González‐Rodríguez**: conceptualization, investigation, and writing; **Tanja Salomaa**: investigation; **Carolina Cabalin**: investigation and resources; **Tuomas Komulainen**: investigation and writing; **Lotta Hiihtola**: investigation; **Laura Kummola**: investigation; **Tero A. H. Järvinen**: supervision and writing; **Ilkka S. Junttila**: conceptualization, supervision, and writing.

## Conflicts of Interest

The authors declare no conflicts of interest.

## Supporting information




**Supporting File: 1** eji70178‐sup‐0001‐SuppMat.pptx.

**Supporting File: 2** eji70178‐sup‐0002‐SuppMat.docx.

## Data Availability

Raw data are available from the corresponding author.
