# Supplementary material for: Dynamic Thymic Stromal Lymphopoietin and Its Receptor Complex Expression on Skin Langerhans Cells in Response to MC903‐induced Skin Inflammation
Source: Eur J Immunol. 2026 Mar 30;56(4):e70178. doi: 10.1002/eji.70178 (PMC13036299; doi:10.1002/eji.70178)
Supplement: Supplementary file 1 — Supporting File: 1 eji70178‐sup‐0001‐SuppMat.pptx. [file EJI-56-e70178-s001.pptx]

## Slide 1
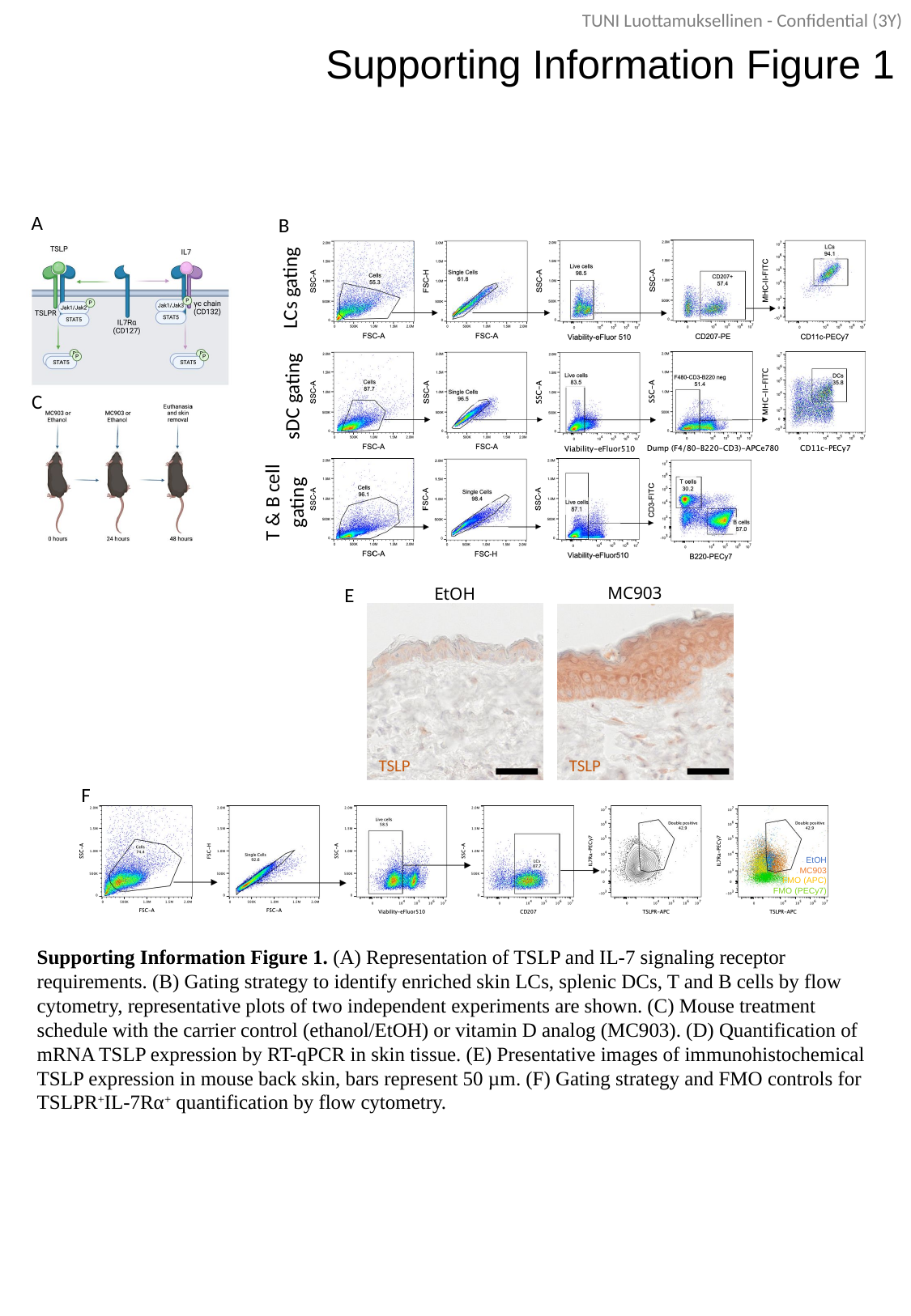

# Supporting Information Figure 1
A
B
LCs gating
sDC gating
C
T & B cell
gating
E
MC903
EtOH
TSLP
TSLP
F
EtOH
MC903
FMO (APC)
FMO (PECy7)
Supporting Information Figure 1. (A) Representation of TSLP and IL-7 signaling receptor requirements. (B) Gating strategy to identify enriched skin LCs, splenic DCs, T and B cells by flow cytometry, representative plots of two independent experiments are shown. (C) Mouse treatment schedule with the carrier control (ethanol/EtOH) or vitamin D analog (MC903). (D) Quantification of mRNA TSLP expression by RT-qPCR in skin tissue. (E) Presentative images of immunohistochemical TSLP expression in mouse back skin, bars represent 50 µm. (F) Gating strategy and FMO controls for TSLPR+IL-7Rα+ quantification by flow cytometry.

## Slide 2
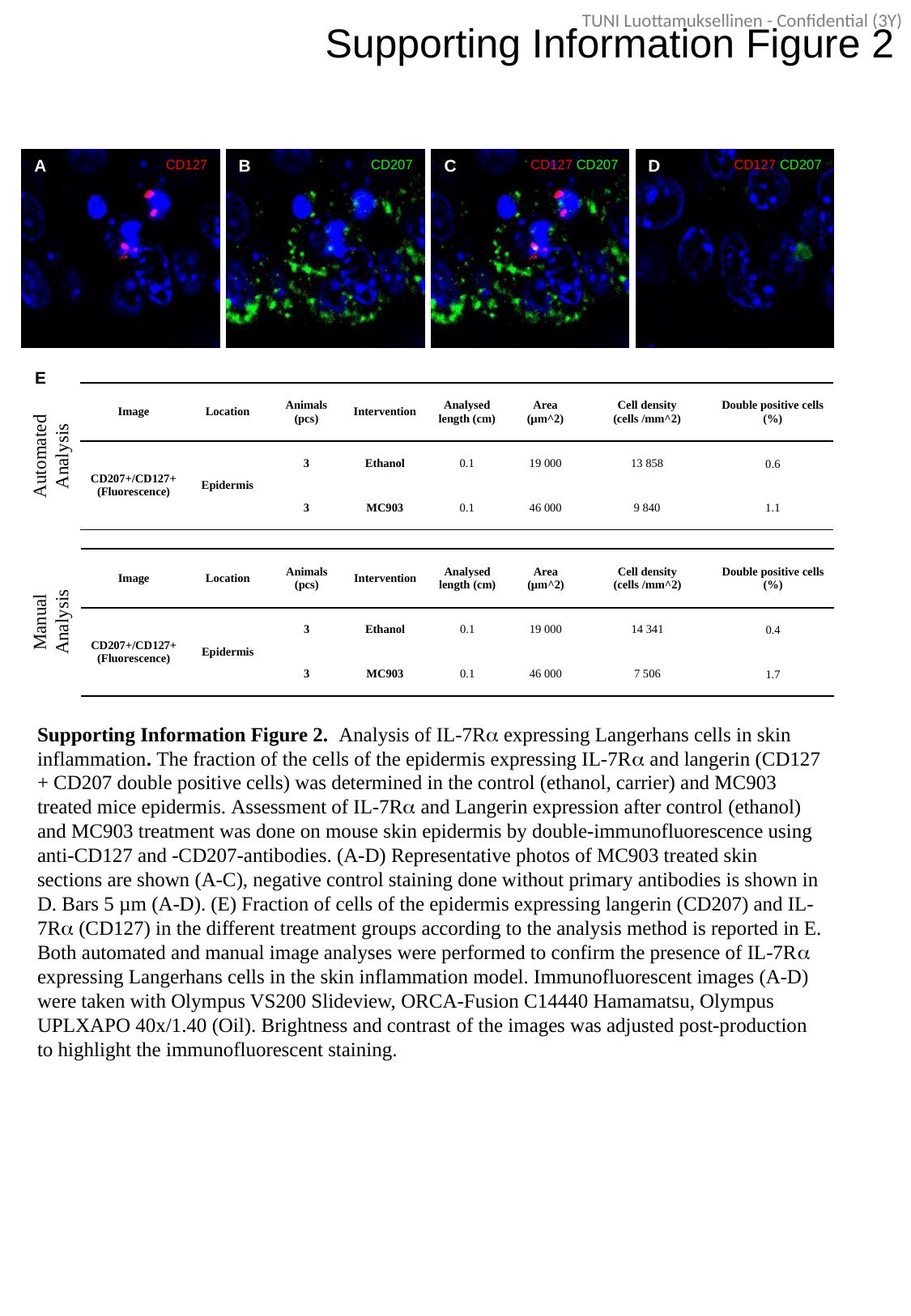

Supporting Information Figure 2
A
B
C
D
CD127
CD207
CD127 CD207
CD127 CD207
E
| Image | Location | Animals (pcs) | Intervention | Analysed length (cm) | Area (µm^2) | Cell density (cells /mm^2) | Double positive cells (%) |
| --- | --- | --- | --- | --- | --- | --- | --- |
| CD207+/CD127+ (Fluorescence) | Epidermis | 3 | Ethanol | 0.1 | 19 000 | 13 858 | 0.6 |
| | | 3 | MC903 | 0.1 | 46 000 | 9 840 | 1.1 |
Automated
Analysis
| Image | Location | Animals (pcs) | Intervention | Analysed length (cm) | Area (µm^2) | Cell density (cells /mm^2) | Double positive cells (%) |
| --- | --- | --- | --- | --- | --- | --- | --- |
| CD207+/CD127+ (Fluorescence) | Epidermis | 3 | Ethanol | 0.1 | 19 000 | 14 341 | 0.4 |
| | | 3 | MC903 | 0.1 | 46 000 | 7 506 | 1.7 |
Manual
Analysis
Supporting Information Figure 2. Analysis of IL-7Rα expressing Langerhans cells in skin inflammation. The fraction of the cells of the epidermis expressing IL-7Rα and langerin (CD127 + CD207 double positive cells) was determined in the control (ethanol, carrier) and MC903 treated mice epidermis. Assessment of IL-7Rα and Langerin expression after control (ethanol) and MC903 treatment was done on mouse skin epidermis by double-immunofluorescence using anti-CD127 and -CD207-antibodies. (A-D) Representative photos of MC903 treated skin sections are shown (A-C), negative control staining done without primary antibodies is shown in D. Bars 5 µm (A-D). (E) Fraction of cells of the epidermis expressing langerin (CD207) and IL-7Rα (CD127) in the different treatment groups according to the analysis method is reported in E. Both automated and manual image analyses were performed to confirm the presence of IL-7Rα expressing Langerhans cells in the skin inflammation model. Immunofluorescent images (A-D) were taken with Olympus VS200 Slideview, ORCA-Fusion C14440 Hamamatsu, Olympus UPLXAPO 40x/1.40 (Oil). Brightness and contrast of the images was adjusted post-production to highlight the immunofluorescent staining.

## Slide 3
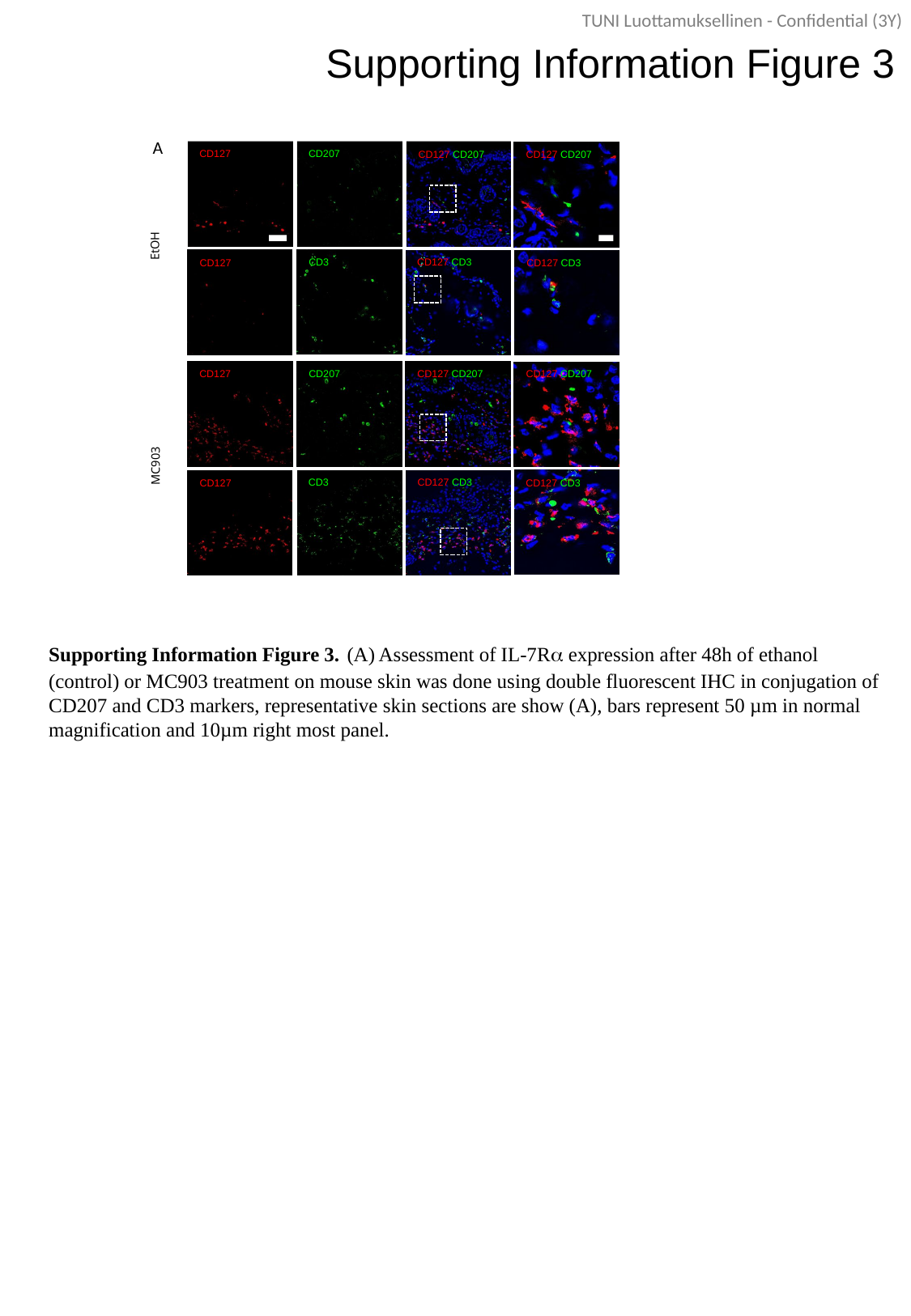

# Supporting Information Figure 3
A
CD207
CD127
CD127 CD207
CD127 CD207
EtOH
CD3
CD127 CD3
CD127
CD127 CD3
CD207
CD127 CD207
CD127
CD127 CD207
MC903
CD127 CD3
CD3
CD127 CD3
CD127
Supporting Information Figure 3. (A) Assessment of IL-7Rα expression after 48h of ethanol (control) or MC903 treatment on mouse skin was done using double fluorescent IHC in conjugation of CD207 and CD3 markers, representative skin sections are show (A), bars represent 50 µm in normal magnification and 10µm right most panel.

## Slide 4
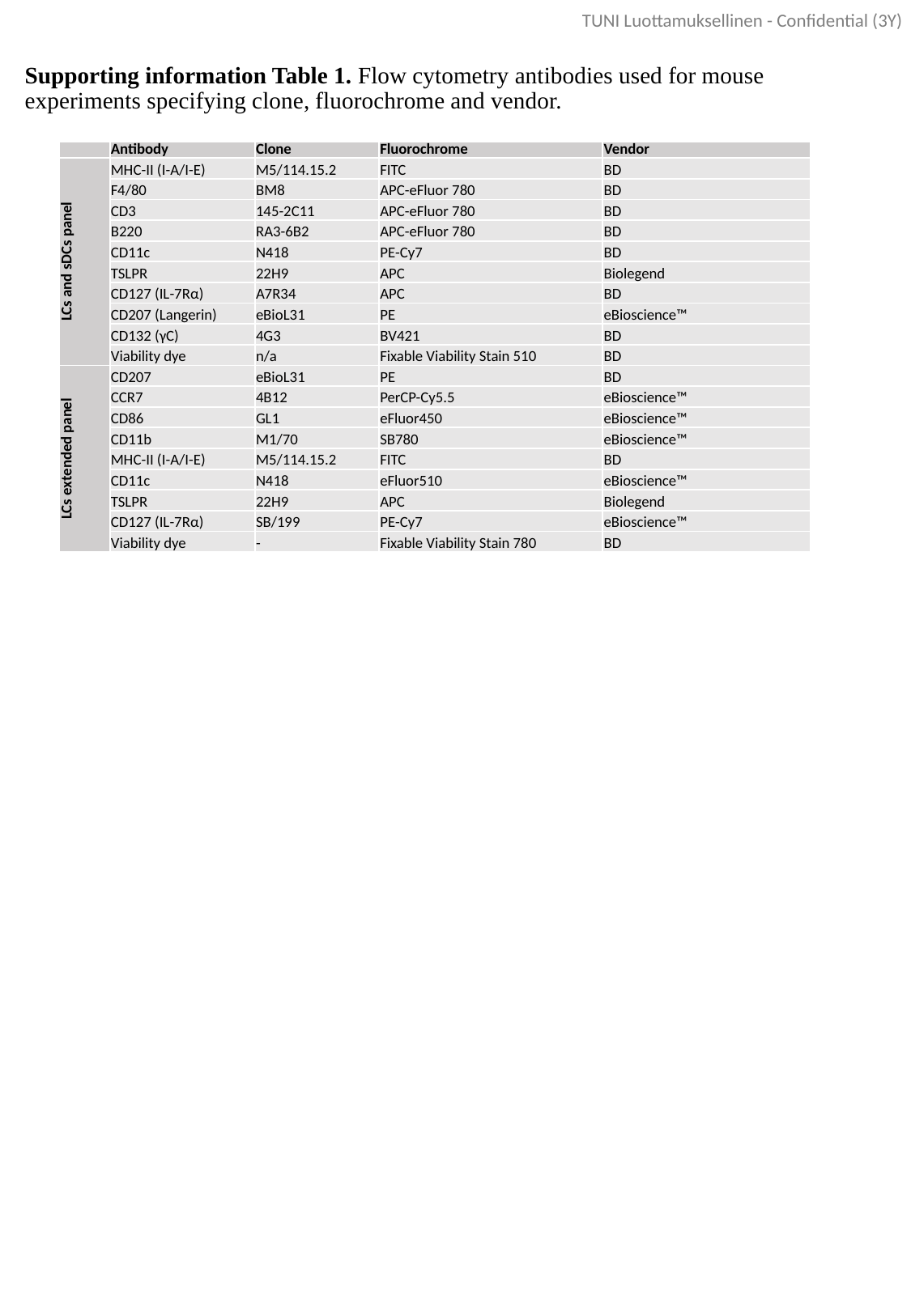

Supporting information Table 1. Flow cytometry antibodies used for mouse experiments specifying clone, fluorochrome and vendor.
| | Antibody | Clone | Fluorochrome | Vendor |
| --- | --- | --- | --- | --- |
| LCs and sDCs panel | MHC-II (I-A/I-E) | M5/114.15.2 | FITC | BD |
| | F4/80 | BM8 | APC-eFluor 780 | BD |
| | CD3 | 145-2C11 | APC-eFluor 780 | BD |
| | B220 | RA3-6B2 | APC-eFluor 780 | BD |
| | CD11c | N418 | PE-Cy7 | BD |
| | TSLPR | 22H9 | APC | Biolegend |
| | CD127 (IL-7Rα) | A7R34 | APC | BD |
| | CD207 (Langerin) | eBioL31 | PE | eBioscience™ |
| | CD132 (γC) | 4G3 | BV421 | BD |
| | Viability dye | n/a | Fixable Viability Stain 510 | BD |
| LCs extended panel | CD207 | eBioL31 | PE | BD |
| | CCR7 | 4B12 | PerCP-Cy5.5 | eBioscience™ |
| | CD86 | GL1 | eFluor450 | eBioscience™ |
| | CD11b | M1/70 | SB780 | eBioscience™ |
| | MHC-II (I-A/I-E) | M5/114.15.2 | FITC | BD |
| | CD11c | N418 | eFluor510 | eBioscience™ |
| | TSLPR | 22H9 | APC | Biolegend |
| | CD127 (IL-7Rα) | SB/199 | PE-Cy7 | eBioscience™ |
| | Viability dye | - | Fixable Viability Stain 780 | BD |

## Slide 5
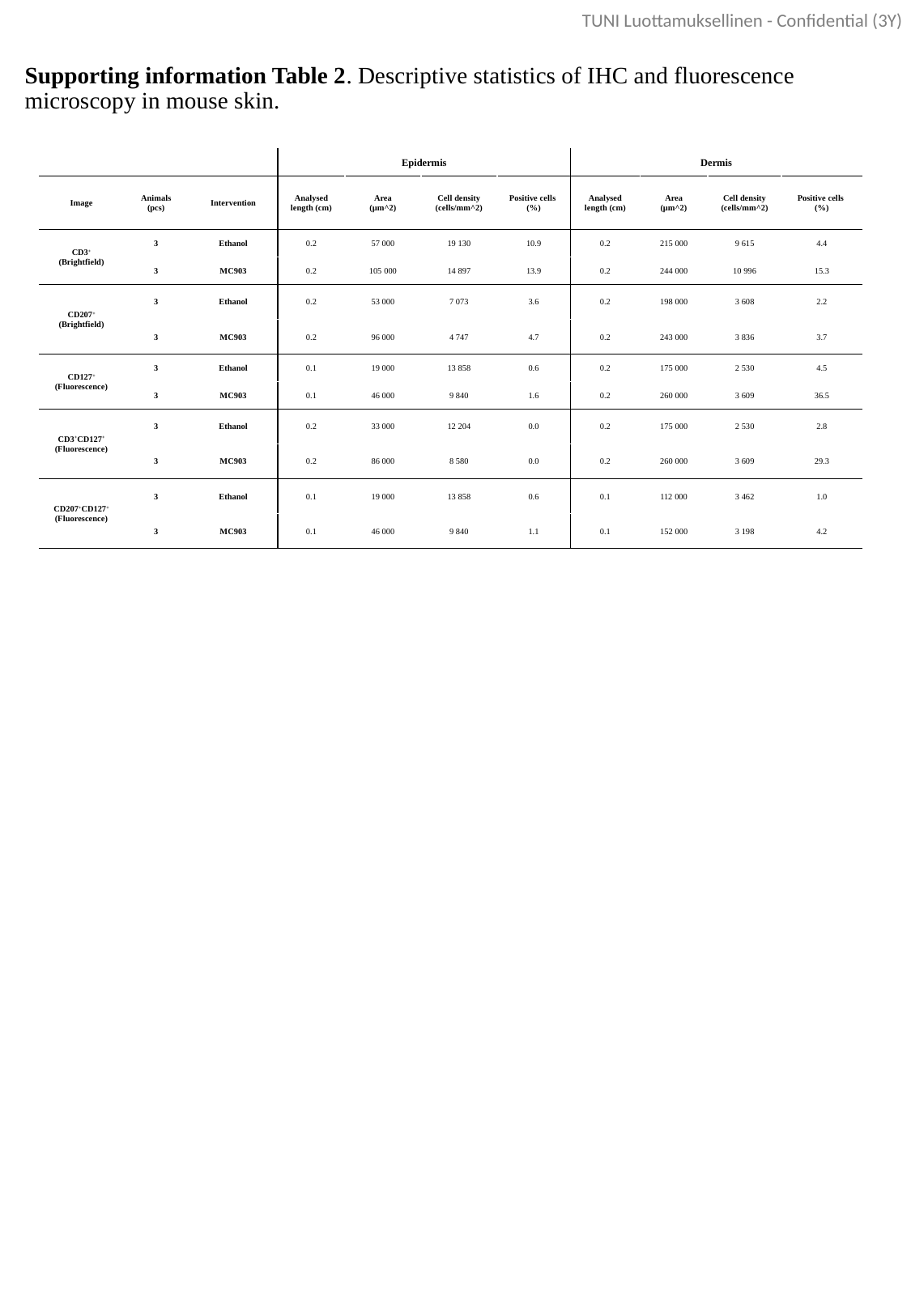

Supporting information Table 2. Descriptive statistics of IHC and fluorescence microscopy in mouse skin.
| ​ | ​ | ​ | Epidermis​ | | | | Dermis​ | | | |
| --- | --- | --- | --- | --- | --- | --- | --- | --- | --- | --- |
| Image​ | Animals​ (pcs)​ | Intervention​ | Analysed​ length (cm)​ | Area​ (µm^2)​ | Cell density​ (cells/mm^2)​ | Positive cells ​ (%)​ | Analysed​ length (cm)​ | Area​ (µm^2)​ | Cell density​ (cells/mm^2)​ | Positive cells ​ (%)​ |
| CD3+​ (Brightfield)​ | 3​ | Ethanol​ | 0.2​ | 57 000​ | 19 130​ | 10.9​ | 0.2​ | 215 000​ | 9 615​ | 4.4​ |
| | 3​ | MC903​ | 0.2​ | 105 000​ | 14 897​ | 13.9​ | 0.2​ | 244 000​ | 10 996​ | 15.3​ |
| CD207+​ (Brightfield)​ | 3​ | Ethanol​ | 0.2​ | 53 000​ | 7 073​ | 3.6 | 0.2​ | 198 000​ | 3 608​ | 2.2​ |
| | 3​ | MC903​ | 0.2​ | 96 000​ | 4 747​ | 4.7​ | 0.2​ | 243 000​ | 3 836​ | 3.7​ |
| CD127+​ (Fluorescence)​ | 3​ | Ethanol​ | 0.1​ | 19 000​ | 13 858​ | 0.6​ | 0.2​ | 175 000​ | 2 530​ | 4.5​ |
| | 3​ | MC903​ | 0.1​ | 46 000​ | 9 840 | 1.6​ | 0.2​ | 260 000​ | 3 609​ | 36.5​ |
| CD3+CD127+​ (Fluorescence)​ | 3​ | Ethanol​ | 0.2​ | 33 000​ | 12 204​ | 0.0​ | 0.2​ | 175 000​ | 2 530​ | 2.8​ |
| | 3​ | MC903​ | 0.2​ | 86 000​ | 8 580​ | 0.0​ | 0.2​ | 260 000​ | 3 609​ | 29.3​ |
| CD207+CD127+​​ (Fluorescence)​ | 3​ | Ethanol​ | 0.1​ | 19 000​ | 13 858​ | 0.6​ | 0.1​ | 112 000​ | 3 462​ | 1.0​ |
| | 3​ | MC903​ | 0.1​ | 46 000​ | 9 840 | 1.1​ | 0.1​ | 152 000​ | 3 198​ | 4.2​ |

## Slide 6
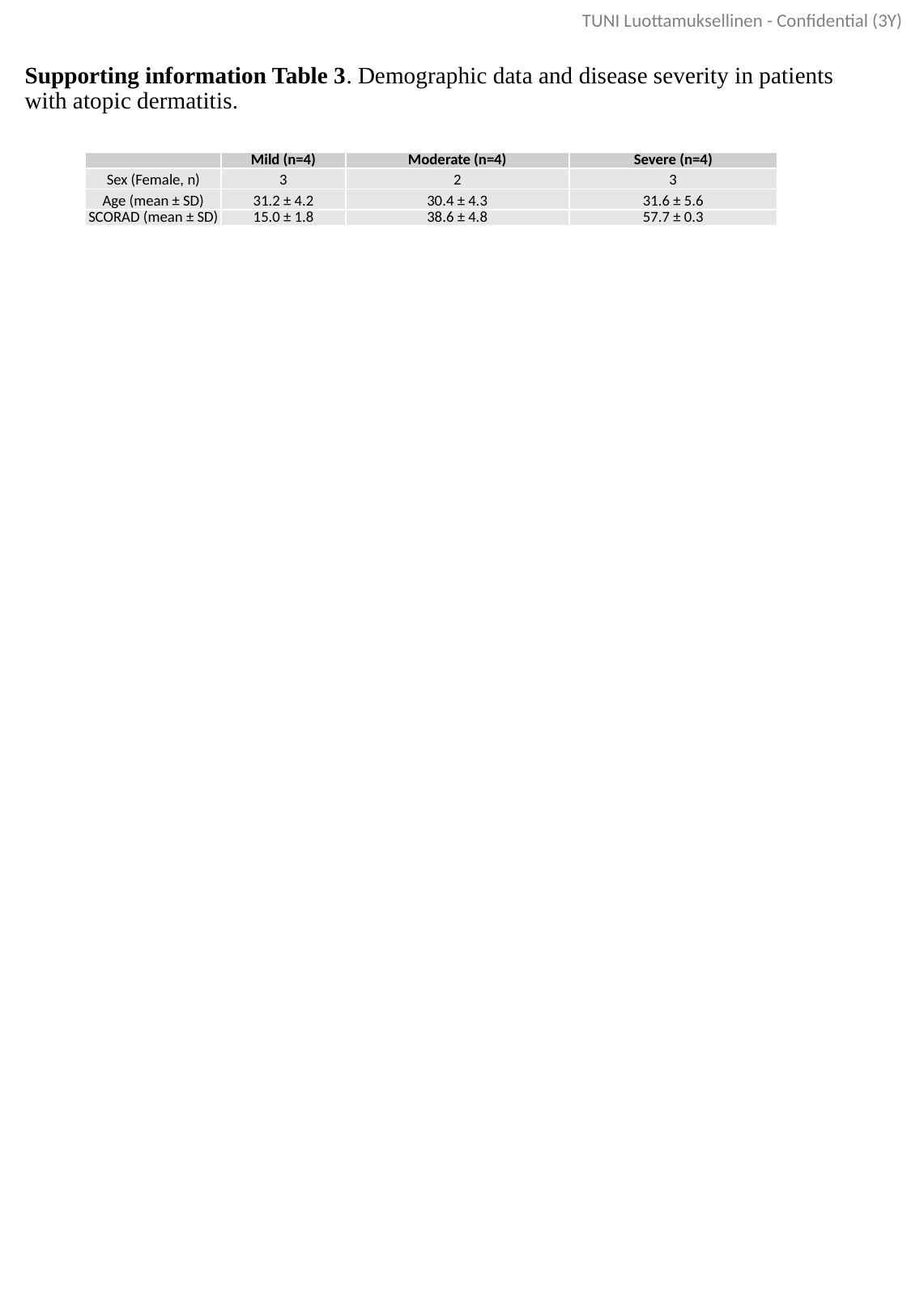

Supporting information Table 3. Demographic data and disease severity in patients with atopic dermatitis.
| | Mild (n=4) | Moderate (n=4) | Severe (n=4) |
| --- | --- | --- | --- |
| Sex (Female, n) | 3 | 2 | 3 |
| Age (mean ± SD) | 31.2 ± 4.2 | 30.4 ± 4.3 | 31.6 ± 5.6 |
| SCORAD (mean ± SD) | 15.0 ± 1.8 | 38.6 ± 4.8 | 57.7 ± 0.3 |
