# Supplementary material for: Dynamic Thymic Stromal Lymphopoietin and Its Receptor Complex Expression on Skin Langerhans Cells in Response to MC903‐induced Skin Inflammation
Source: Eur J Immunol. 2026 Mar 30;56(4):e70178. doi: 10.1002/eji.70178 (PMC13036299; doi:10.1002/eji.70178)
Supplement: Supplementary file 2 — Supporting File: 2 eji70178‐sup‐0002‐SuppMat.docx. [file EJI-56-e70178-s002.docx]

**Dynamic thymic stromal lymphopoietin (TSLP) and its receptor complex expression on skin Langerhans cells in response to MC903-induced skin inflammation**

***Authors***

Martín I. González-Rodríguez1, Tanja Salomaa1, Carolina Cabalin2-4, Tuomas Komulainen1, Lotta Hiihtola1, Laura Kummola1, Tero A.H. Järvinen1,5, Ilkka S. Junttila1,6-7

Affiliations:

1. Faculty of Medicine and Health Technology, Tampere University(TUNI)
2. InverSkin SpA, Santiago, Chile
3. Department of Infectious Diseases and Pediatric Immunology, School of Medicine, Santiago, Pontifical Catholic University of Chile, Santiago, Chile
4. Department of Dermatology, Weill Cornell Medicine, New York, NY, USA.
5. Department of Orthopaedics and Traumatology, TAUH, Tampere, Finland.
6. Fimlab Laboratories, Tampere, Finland
7. NordLab, Oulu, Finland.

***Correspondence***: Ilkka Junttila, Arvo-Ylpönkatu-34, 33520, Tampere, Finland. [Ilkka.junttila@tuni.fi](mailto:Ilkka.junttila@tuni.fi)

***Keywords***: Langerhans cells, TSLP

***Abbreviations***: Thymic Stromal Lymphopoietin; TSLP, Langerhans cell; LC, Atopic dermatitis; AD, Interleukin;IL, MC903; Calcipotriol, Stat; signal transducer and activator of transcription, t-distributed stochastic neighbor embedding analysis; tSNE

***Materials and methods***

*Animals & Ethical approval*. Female C57BL/6J (Jax line 00664) mice from The Jackson Laboratory (Bar Harbor, ME), aged 7-10 weeks at the beginning of the experimental procedure, were used in this study. Ethical approval for all animal work was provided by the Regional State Administrative Agency (AVI, animal permit number ESAVI/275/04.10.07/2018). Animals were housed in individual ventilated cages (IVC) in Tampere University animal facility. The animals from different groups were fed with the same food and drinking *water ad libitum*. At the final experimental day, mice were euthanized in CO_2_ chamber (Pre-Set CO2 System-1 Chamber, AS044AX, Able Scientific, Perth, Australia) and organs were subsequently collected for analysis.

All animal experiments, including flow cytometry and quantitative histological analyses were carried out by adhering to ARRIVE 2.0 guidelines (1).

*Topical vitamin D analogue administration. treatment.* Vitamin D analogue (MC903, Calcipotriol, Sigma) was dissolved in 100% ethanol and topically applied as previously described in 1cm^2^ of the back skin (10 nmol in 100 µl per animal).

*Flow cytometric analysis*. Spleen were collected from animals to enrich splenic dendritic cells. Single-cell suspensions were prepared by mechanical dissociation of the organs by first mincing them and then treated with Liberase DL (Roche Molecular Systems, Inc.) and DNase (Qiagen. US) for 30 mins at 37°C. Later, spleen parts were smashed through a 70 µm cell strainer (Fisher Scientific, US) into PBS-/- (pH 7.2, Thermo Fisher Scientific, Waltham, MA) supplemented with 1% heat-inactivated FBS (Gibco, Thermo Fisher Scientific, US) and 2mM EDTA. After centrifugation, RBCs were lysed with 1 min ACK (Lonza, Basel, Switzerland) treatment and then washed with PBS containing 1% FBS and 2mM EDTA. Splenic dendritic cells were enriched using the Pan Dendritic Cell Isolation Kit (Miltenyi Biotec). Langerhans cells were enriched from the back skin of mice using the Epidermis Dissociation Kit (Miltenyi Biotec) together with the Epidermal Langerhans Cell MicroBead Kit (Miltenyi Biotec). Single-cell suspensions were resuspended in PBS and stained with viability dye FVS510 (Becton Dickinson Biosciences, Franklin Lakes, NJ) for 20 minutes, RT, followed by washing with FBS buffer. Fc receptors were blocked with Rat Anti-Mouse CD16/CD32 antibodies (Mouse BD Fc Block™, BD) for 5 min at 4°C before staining. Antibodies for flow cytometry were purchased from BD or Thermo Fisher Scientific (Supplementary Table 1). Staining was performed at 4°C for 20 min and cells were washed twice with FBS buffer before analyzing them. Dimensional reduction analysis (t-SNE) was performed using FlowJo software including down-sampling, sample concatenation and unsupervised clustering by FlowSOM. All samples were run with CytoFLEX S Flow Cytometer (Beckman Coulter life sciences) and analyzed with FlowJo software version 10.8.1 (BD).

*RNA extraction and RT-qPCR analysis*. RNA extraction for skin samples were disrupted using CKMix tubes (VWR, Precellys®) containing 30-40mg of skin tissue and 1mL of TRIzol™ Reagent (Invitrogen, Thermo Fisher scientific). Shortly, tubes were homogenized using Precellys Tissue homogenizer (Bertin) following 4 times rounds of 30s shakes at 6500 RPM with 30s intervals in ice. Samples were centrifuged at 12000g for 10 min at 4°C and transferred to 1,5 mL tubes. Homogenized sample was incubated for 5 min at room temperature, 0.2 mL of chloroform was added and vortexed for 15s. Tubes were centrifuged at 12000g for 15 mins at 4°C and clear phases were transferred to new 1,5mL tubes for further RNA extraction following RNeasy Mini Kit (Qiagen). Then, RNA extraction was conducted following the RNeasy Mini Kit (Qiagen). Using iScript™ cDNA Synthesis Kit (Biorad, Hercules, CA) cDNA synthesis from RNA samples was performed. Gene expression was assessed using RT-qPCR using Sybrgreen and ABI QuantStudio 12K Flex System (Thermo Fisher Scientific).

*Double-Immunofluorescence.* Double-immunofluorescence staining was performed on 5 µm thick paraffin sections. For double-immunofluorescence, different antigen retrieval methods were first tested for each antibody to identify the ideal method. The antigen retrieval was carried out by immersing the slides into 10mM TRIS-HCl, 1mM EDTA (pH 9) in the standard microwave oven (350 W) (2,3). Was carried out sequentially using the following primary antibodies: rabbit monoclonal Anti-mouse Langerin (1:100, EPR24685-12, Abcam), Rat anti-mouse IL7Rα /CD127 (1:100, 14-1271-82, Invitrogen) and rabbit anti-human CD3 (1:500, A0452, Dako) (4). 2.5% BSA was used as a blocking agent and 3% hydrogen peroxidase treatment of the tissue sections was carried out. The secondary antibodies used were Alexa Fluor 647-conjugated fab fragment goat anti-rat IgG (1:100, 112-607-003, Invitrogen) and HRP-conjugated goat anti-rabbit antibody followed by AlexaFluor 488 Tyramide SuperBoost according to the manufacturer´s instructions (B40943, Invitrogen), ending by stop solution and DAPI (MBD0015, Sigma). The samples were mounted with Fluoroshield™ with 1,4-Diazabicyclo[2.2.2]octane mounting medium (Sigma-Aldrich).

*Immunohistochemical (IHC). IHC* staining was performed on 5 µm thick paraffin sections. IHC was carried out using appropriate antigen retrieval method for each staining. The following primary antibodies were used for IHC (according to the manufacturer’s instructions): TSLP Receptor Polyclonal (1:200, PA5-47979, Invitrogen) and TSLP Polyclonal (1:700, PA520320, Invitrogen). The blocking reagents used were Peroxidase-Blocking Solution (S202386-2) (5). The secondary antibodies used were Goat-on-Rodent HRP-Polymer for TSLPr and Rabbit on rodent HPP (RMR622H) for TSLP, followed by peroxidase reactive chromogen DAB (K3465, Dako) and Papanicolaou′s solution 1a Harris′ hematoxylin solution (1.09253,Merck). The samples were mounted with Fluoroshield™ with 1,4-Diazabicyclo[2.2.2]octane mounting medium (Sigma-Aldrich).

*Virtual microscopy and quantitative manual and automated analyses of the IHC and the immune-fluorescence***.** All the histological sections were scanned to digital images using Olympus VS200 Slideview ORCA-Fusion C14440 Hamamatsu, Olympus UPLXAPO 40x/1.40 (Oil) or Olympus VS-264C, Olympus UPlanXApo 20x/0.80, WD 0.60 mm (Air). Quantitative analyses were done with QuPath version 0.4.3, opensource pathology image analysis software (6). From each 1.1 -1.5 cm long skin sample 0.1 cm or 0.2 cm long Region-of-interests (ROI) with the most prominent reaction were determined manually by researcher blinded on the treatment. The manual drawing of the ROI sincluded the entire dermis and epidermis if necessary. Positively staining cells (fraction) of all detected cells were quantified with the automated image analysis tools as described previously (7).The fraction of CD207+CD127+ double positive cells in the epidermis was also verified with manual analysis. The thresholds for positivity were selected manually for each sample in each antibody group. For all double-immunofluorescence analyses, scanning and quantification processes were performed in blinded fashion. All the samples were coded during tissue harvesting and the code was decrypt only after the completion of the analyses. The researcher was blinded to the treatment allocation during the analysis.

*Study design and collection of skin samples*

A pilot case-control study was conducted to collect tape strips and biopsies from lesional and non-lesional skin from patients with atopic dermatitis. Inclusion criteria were patients with AD diagnosed by Hanifin and Rajka criteria who had SCORAD ≥10 (i.e., mild–severe AD)(8). Demographic and clinical characteristics of participants were registered, including reports of physician-diagnosed atopic dermatitis SCORAD index. AD severity was categorized as mild

if SCORAD<25, moderate if SCORAD 25–49.9, and severe if SCORAD resulted≥50.

Non-lesional and lesional skin samples were obtained by the tape stripping technique using 15 D-Squame (Cuderm) standard sampling discs on the same site. These samples were then stored at -80°C until analysis. RNA was extracted from sampling discs using TRIZOL from Ambion (Invitrogen). The RNA quantification was made by Qubit 4.0 (Invitrogen) and cDNA was obtained with a high-capacity cDNA reverse transcription kit (Applied Biosystems). Quantitative PCR (qPCR) of cDNA was performed with TaqMan probes (Applied Biosystem) for gapdh (Hs02758991) as endogenous control, tslp (Hs00263639) and tslpr (Hs00845692_m1, also named CRLF2 in human) in the QuantStudio6 of Applied Biosystems. Gene expression was assessed as fold change according to the 2^−ΔΔCt^ method.

Also, we collected a 3 mm punch biopsy from the same site. IHC was performed on frozen skin sections using purified anti-human antibodies for TSLPR (anti-CRLF2 antibody, ab109626, Abcam) and IL7a (Anti-CD127 antibody [EPR23747-333], ab259806, Abcam). Images were captured using a trinocular microscope at a 20X magnification. Positive cells per mm were quantified by using ImageJ software (version 1.42, National Institutes of Health, Bethesda, Md).

*Statistical analysi*s. Normal distribution was assessed for each data set using Anderson-Darling tests or Shapiro-Wilk test when data sets did not fulfill the requirements (alpha=0.05). Parametric data were analyzed using a One-way ANOVA (alpha=0.05, two-tailed), with Tukey post-test comparison. Non-parametric data were analyzed using the Kruskal-Wallis multiple comparison test with Dunn’s correction. All immunological data sets were analyzed using GraphPad Prism 8.0 (GraphPad Inc., USA).

***Data availability statement***

Raw data available from corresponding author.

***Conflict of interest disclosure***

The authors declare no conflicts of interest.

***Ethics approval statement for human and/or animal studies***

Ethical approval for all animal work was provided by the Regional State Administrative Agency (AVI, animal permit number ESAVI/275/04.10.07/2018). Animals were housed in individual ventilated cages (IVC) in the Tampere University preclinical testing facility. All animal experiments, including flow cytometry and quantitative histological analyses, were carried out by adhering to ARRIVE 2.0 guidelines (1). The written informed consent of all participants was obtained at the beginning of the study.

***Author contributions***

**Author Contributions statement:** MGR Conceptualization, Investigation, Writing, TS Investigation, CC Investigation, Resources, TK Investigation, Writing, LH Investigation, LK Investigation, TAHJ Supervision, Writing, ISJ Conceptualization, Supervision, Writing.

***REFERENCES***

1. Sert NP du, Ahluwalia A, Alam S, Avey MT, Baker M, Browne WJ, et al. Reporting animal research: Explanation and elaboration for the ARRIVE guidelines 2.0. PLOS Biology. 2020 Jul 14;18(7):e3000411. doi:10.1371/journal.pbio.3000411

2. De Rossi G, Vähätupa M, Cristante E, Arokiasamy S, Liyanage SE, May U, et al. Pathological Angiogenesis Requires Syndecan-4 for Efficient VEGFA-Induced VE-Cadherin Internalization. Arteriosclerosis, Thrombosis, and Vascular Biology. 2021 Apr;41(4):1374–89. doi:10.1161/ATVBAHA.121.315941

3. Vähätupa M, Prince S, Vataja S, Mertimo T, Kataja M, Kinnunen K, et al. Lack of R-Ras Leads to Increased Vascular Permeability in Ischemic Retinopathy. Invest Ophthalmol Vis Sci. 2016 Sep 19;57(11):4898. doi:10.1167/iovs.16-19212

4. Komulainen T, Ylitörmä M, Hietanen KE, Järvinen J, Junttila IS, Kaartinen IS, et al. Anti-Inflammatory Macrophages Are Recruited to the Keloids that Obtain Response to Intralesional Injection Therapies: Before and After Treatment Biopsies from a Double-Blinded, Randomized, Controlled Trial. J Invest Dermatol. 2025 Nov 4;S0022-202X(25)03519-5. doi:10.1016/j.jid.2025.10.597 PubMed PMID: 41197764.

5. Maldonado H, Savage BD, Barker HR, May U, Vähätupa M, Badiani RK, et al. Systemically administered wound-homing peptide accelerates wound healing by modulating syndecan-4 function. Nat Commun. 2023 Dec 6;14(1):8069. doi:10.1038/s41467-023-43848-1

6. Bankhead P, Loughrey MB, Fernández JA, Dombrowski Y, McArt DG, Dunne PD, et al. QuPath: Open source software for digital pathology image analysis. Sci Rep. 2017 Dec 4;7(1):16878. doi:10.1038/s41598-017-17204-5

7. Komulainen T, Ylitörmä M, Hietanen KE, Järvinen J, Junttila IS, Kaartinen IS, et al. Anti-Inflammatory Macrophages Are Recruited to the Keloids that Obtain Response to Intralesional Injection Therapies: Before and After Treatment Biopsies from a Double-Blinded, Randomized, Controlled Trial. J Invest Dermatol. 2025 Nov 4;S0022-202X(25)03519-5. doi:10.1016/j.jid.2025.10.597 PubMed PMID: 41197764.

8. Hanifin JM, Rajka G. Diagnostic Features of Atopic Dermatitis. Acta Derm Venereol. 1980 Nov 11;60:44–7. doi:10.2340/00015555924447
